# Dr. Isaac Parrish on the Use of the Iodide of Potassium in Ophthalmic Diseases, with Cases

**Published:** 1842-04-16

**Authors:** Isaac Parrish

**Affiliations:** One of the Surgeons to the Wills Hospital; Philada.


### MEDICAL EXAMINER.

NEW SERIES.

No. 16.] PHILADELPHIA, APRIL 16, 1842. [Vol. I.

*On the Use of the Iodide of Potassium in Ophthalmic Diseases, with Cases.* By Isaac Parrish, M. D., one of the Surgeons to the Wills Hospital.

The value of the iodide of potassium, as a remedy in certain diseases of the eye, which have a constitutional origin, or are closely allied to a scrofulous or cachectic condition of the general system, is beginning to attract attention. During a recent term of service at the Wills Hospital, an opportunity occurred to me of employing the remedy in some cases of this description; and, although the number of patients was too limited to warrant a general conclusion as to its powers, yet the results were so striking and satisfactory, as to create a strong impression in its favour, especially when viewed in connection with the concurrent testimony of others.

If this article could be safely employed as a substitute for mercury in many diseases of the eye, in which we have been accustomed to rely upon that potent medicine; or, if it will produce a strong alterative impression, in cases where an enfeebled or irritable condition of the constitution renders a resort to mercury hazardous, it will constitute a most valuable auxiliary in the treatment of a numerous class of cases, which are exceedingly difficult of cure.

The dose in which we administered the article at the Wills Hospital, was from two to six grains, three times daily, in a table-spoonful of the compound syrup of sarsaparilla. The selection of the latter article as a vehicle for the iodide, was prompted by the experience at the Pennsylvania Hospital, and constitutes, perhaps, an important item in the treatment.

The two following cases have been prepared for publication, from notes furnished me by Dr. Hollingsworth, the resident physician of the hospital. They are selected from several others, as presenting the most decided influence of the remedy under circumstances somewhat embarrassing.

*Case.* Wm. Boyle, aged 23 years, was admitted into the Wills' Hospital in the latter part of the Eleventh month, (Nov.) 1841, for granular ophthalmia. He had been suffering with inflamed eyes for four months prior to his admission, for which he had been treated on a rigid antiphlogistic plan, with the continued application of emollients to the eyes.

Soon after his entrance into the hospital the inflammation of the conjunctiva became more acute, (probably from exposure in travelling;) this was relieved by local depletion from the temples, cooling lotions, &c.; after which solid sulphate of copper was applied to the inner surface of the lids; he was placed on a good diet; took syrup of the iodide of iron, chamomile decoction, &c., under which treatment the eyes rapidly improved. He soon after suffered from another relapse, attended with inflammation and ulceration of the cornea, severe circumorbital pain, with a feeble pulse, and pallid countenance, from which he was again in a great measure relieved, under the treatment of Dr. Hays. It was not long, however, before he was again seized more violently than before, and when I took charge of the wards, at the commencement of the present year, his condition was quite deplorable.

He was suffering from deep seated pain in the head and around the orbit, aggravated at night, and preventing sleep, except under the influence of powerful anodynes; the eyes were highly injected, the sclerotic coat, cornea and iris being involved. He had photophobia to a great degree, and very little vision. The constitutional symptoms were equally discouraging. The pulse was feeble, skin cool and relaxed, and countenance dejected. Under the use of a pill, composed of a grain of sulphate of quinine, and half a grain of the extract of cicuta three times daily, with counter irritation to the nape of the neck, anodynes, nutritious diet, &c., some amendment took place. This, however, was of short duration, all the symptoms returning with increased severity. In the early part of the Second month, (Feb.) erysipelas appeared around the eyelids, and rapidly extended over the face with alarming constitutional symptoms, as delirium, furred tongue, feeble and rapid pulse, &c. The carbonate of ammonia, in doses of five grains, was given with great advantage, together with beef tea, oysters, &c. As the erysipelatous eruption declined, the pain in the head diminished, and the condition of the eyes improved, but still the circumorbital pain continued violent, especially at night. A slough was extending on the cornea of the right eye; great intolerance of light, with injection of the vessels of the conjunctiva and cornea, and contraction of the pupil still existed. The strength of the patient was exceedingly reduced; his appetite poor; expression of the countenance haggard and distressed; features contracted; skin relaxed and pallid; anodynes produced but little comfort; the stomach was irritable and rejected tonics, and I entertained strong fears that vision would be greatly impaired, if not lost, by the still active disease of the eyes. It was under these circumstances that the iodide of potassium was resorted to. It was first given in doses of two grains three times daily, in a table-spoonful of the compound syrup of sarsaparilla.

An improvement was manifest in less than forty-eight hours, which induced us to increase the dose to five grains.

Under this treatment the pain vanished in a short time, the patient slept soundly without anodynes, his strength and appetite rapidly improved, and for the first time since his admission, he was unaffected by changes of weather.

The application of a solution of the nitrate of silver to the eyes, together with the improvement of the general symptoms, produced a corresponding change in the local inflammation; and when I left the house, his case was in every respect promising, but little doubt existing that he would recover the use of his eyes. He was still taking the remedy in doses of six grains three times daily, and had not suffered from pain in the head since he commenced it.

The case of James Dougherty was in some respects analogous to the above. This man was admitted in Tenth month, (Oct.) 1841, for chronic conjunctivitis, which had existed for a considerable time. He complained of a steady dull pain in the side of the head, over the parietal bone, which extended at times over the scalp, and was often very acute. The pain was aggravated at night, was attended with excessive intolerance of light, and injection of the conjunctiva, sclerotic, and cornea. The patient was greatly affected by changes of weather; slept but little even after anodynes, and laboured under great constitutional irritation.

The local applications generally employed at the hospital in similar cases, were of no avail; nor did any treatment appear to produce permanent or decided benefit. The dull pain on the side of the scalp was constantly complained of, and the patient was afflicted with frequent paroxysms of the most acute suffering, which extended over the cranium, and around the orbits. After trying a great variety of remedies for more than two months, I was induced, from the success attending Boyle's case, to resort to the same remedy in this case. Three grains of the iodide of potassium were given three times daily in the syrup of sarsaparilla, and all other remedies laid aside. The change which ensued was surprising. In two or three days, the pain which had been so constantly present for several months was greatly alleviated, so that the patient was able to sleep without anodynes. The dose was now increased to five grains, under which the appetite improved, and the general health of the patient seemed to undergo a revolution. The pain disappeared altogether; the injection of the eyes, and the intolerance of light were rapidly diminishing; and when I left the patient under the care of my successor, Dr. Fox, the prospect of his speedy recovery appeared very encouraging.

*Remarks.*—The most striking effect which the iodide appeared to produce in these cases, was its influence on the severe neuralgic pain, from which the patients had suffered so long and so intensely. The remedy seemed to put a period to this in a very short time, and the relief was permanent, placing the patient beyond the influence of those changes of temperature which so often induce or aggravate this peculiar affection. As a consequence of this immunity from suffering, tranquil sleep was enjoyed, the appetite and strength returned, and the local inflammation rapidly subsided.

The remedy was tried in several other cases of strumous inflammation of the eye, in all of which its effect was happy, except in one instance. That was the case of a young woman with scrofulous iritis, whose disease had resisted a great variety of treatment during several months, and in which I had strong hopes of effecting a change by the iodide. It produced in this instance severe vomiting and purging in doses of three grains, and could not be borne even in two grain doses.

In the case of a young woman with scrofulous conjunctivitis, in which the cornea and iris were slightly involved, secondarily, I relied altogether on the iodide in five grain doses, without any external applications, except a few leeches to the temple, in the commencement of the attack. The improvement in this case was much more rapid and steady than is usual in this form of disease.

*Philada.*, Fourth mo. 10th, 1842.

